# AMP-activated protein kinase: a therapeutic target in intestinal diseases

**DOI:** 10.1098/rsob.170104

**Published:** 2017-08-23

**Authors:** Xiaofei Sun, Mei-Jun Zhu

**Affiliations:** 1 School of Food Science, Washington State University, Pullman, WA 99164, USA; 2 School of Food Science, University of Idaho, Moscow, ID 83844, USA

**Keywords:** AMPK, absorption, barrier function, colorectal cancer, intestinal health, intestinal inflammation

## Abstract

Adenosine monophosphate (AMP)-activated protein kinase (AMPK), a highly conserved energy sensor, has a crucial role in cardiovascular, neurodegenerative and inflammatory diseases, as well as in cancer and metabolic disorders. Accumulating studies have demonstrated that AMPK activation enhances paracellular junctions, nutrient transporters, autophagy and apoptosis, and suppresses inflammation and carcinogenesis in the intestine, indicating an essential role of AMPK in intestinal health. AMPK inactivation is an aetiological factor in intestinal dysfunctions. This review summarizes the favourable outcomes of AMPK activation on intestinal health, and discusses AMPK as a potential therapeutic target for intestinal diseases.

## 1. Introduction

The intestine is the longest and largest vital epithelial organ. Its major functions are absorbing nutrients from food and establishing selectively permeable barriers against the external environment [1]. To execute these functions, the intestinal epithelium needs to form a barrier, which depends on a well-balanced cellular homeostasis, orchestrated by a delicate interaction and balance among differentiation, self-renewal, proliferation and the intestinal ecosystem [2]. Disruption of the balance in intestinal homeostasis causes enteritis and colitis, leading to malnutrition, diarrhoea, weight loss, constipation and fatigue [3,4]. Losses in intestinal homeostasis are associated with a broad range of pathological changes including metabolic disorders, inflammatory and autoimmune diseases, and cancers [5,6].

Adenosine monophosphate (AMP)-activated protein kinase (AMPK), a serine/threonine kinase, is evolutionarily conserved from yeast to mammals. As an energy sensor, AMPK is activated by upstream enzymes when the cellular ratio of AMP to adenosine triphosphate (ATP) is elevated due to nutrient deprivation [7]. After activation, AMPK phosphorylates downstream substrates to promote catabolism and impede anabolism, leading to ATP production and energy restoration [8,9]. AMPK activity can be altered owing to numerous physiological factors, such as hormones, cytokines and dietary nutrients, as well as pathological conditions such as obesity, chronic inflammation and type II diabetes [10]. Thus, AMPK activation can act as a therapeutic agent to treat various metabolic diseases [11,12]. Furthermore, the function of AMPK on the metabolism of liver and skeletal muscle has been well studied and documented [13,14]. AMPK also has a crucial role in regulating cellular development, such as adipogenesis [15], myogenesis [16] and osteogenesis [17].

Accumulating evidence supports the beneficial effects of AMPK on gut health, such as enhancing intestinal absorption [18], improving barrier function [19], suppressing colorectal carcinogenesis [20], and reducing intestinal inflammation [21] and metabolic-related disease [22]. Conversely, AMPK is inhibited under diabetic and obese conditions, which is correlated with impaired intestinal barrier function [23]. The inhibition of AMPK under pathological and physiological states has been comprehensively discussed in a previous review [10]. In this review, we summarize the regulatory role of AMPK in intestinal health and diseases, which links the important energy sensor to the maintenance of intestinal homeostasis.

## 2. AMPK and its regulators in the intestine

Intestinal mucosa contains the epithelial layer, the lamina propria, consisting mainly of connective tissue, and the muscularis mucosae layer, made of smooth muscle. AMPK is mainly located at the apical part of the villus epithelium in human adult jejunum [24]. AMPK is a heterotrimer, consisting of α catalytic subunits, and *β* and *γ* regulatory subunits. AMPK is activated by phosphorylation at Thr 172 of the *α* subunit by upstream kinases, such as liver kinase B1 (LKB1) and calmodulin-dependent protein kinase kinase (CaMKK) [25,26]. The binding of AMP to the *γ* subunit causes conformational changes which facilitate its phosphorylation by AMPK activators, such as LKB1 [27]. The *α* and *β* subunits each have two isoforms (α1 and α2, and β1 and β2), while the γ subunit has three isoforms (γ1, 2 and 3) [7]. The different heterotrimeric complexes display tissue specificity [24]. The AMPK α1/β2/γ1 complex is more abundant in differentiated intestinal epithelial cells [24]. Our recent study found that AMPK α1 deletion in intestinal epithelium suppresses intestinal differentiation in mouse jejunum with reduced mucosal height and villin content [19]. No change of epithelial architecture occurs in AMPKα2-deleted mice [28], which might be due to the predominance of the α1 subunit in intestinal epithelium. The layers of connective tissue and smooth muscle tend to be thinner [28], which is probably due to the expression of the α2 subunit in non-epithelial cells. In addition, α1 is expressed during the initial stages in myogenesis, while α2 becomes dominant in differentiated myogenic cells [29], illustrating the tissue-specific expression of AMPK α isoforms.

AMPK is linked to the beneficial effects of nutraceutical or pharmacological compounds in intestinal health (table 1). 5-Aminoimidazole-4-carboxamide ribonucleotide (AICAR) is commonly used as a pharmacological activator for AMPK. It triggers AMPK activation through conversion into ZMP (Z refers to imidazole), an AMP analogue mimicking the AMP effect [62]. As expected, AMPK is activated in Caco-2 cells in response to AICAR treatment [24]. It has been shown that AICAR promotes intestinal glucose transportation [63] and barrier function [19], and inhibits infiltration of inflammatory cells [33]. Another pharmacological compound, metformin, a dimethyl-biguanide, is a common anti-diabetic drug [64,65]. Metformin indirectly activates AMPK by inhibiting mitochondrial complex I in the respiratory chain to increase the AMP : ATP ratio [66]. In response to metformin, the phosphorylation of AMPK and its specific substrate acetyl-CoA carboxylase (ACC) increased 10-fold and 5-fold, respectively, in Caco-2 cells [24]. Metformin enhances intestinal glucose transportation [46] and inhibits inflammatory cytokines [67] and colitis [21]. Furthermore, microbial metabolite butyrate and other extracts from plants improve intestinal barrier function [38], suppress peptide transportation [42] and induce apoptosis in Caco-2 cells, associated with enhanced AMPK signalling. Though the mechanisms responsible for AMPK activation remain poorly defined, these plant-origin metabolites might inhibit mitochondrial function, including complex I in the respiratory chain and F1 ATP synthase, to increase the AMP : ATP ratio [63,68].

**Table 1.** Compounds targeting AMPK pathways in the intestine.

experimental setting	compounds	functions	references
HCT116; HT-29; LoVo cells	adiponectin	inhibits cyclin E and cell growth; promotes p21, p27, glucose utilization and fatty acid oxidation	[30,31]
mice jejunum	AICAR	inhibits SGLT1; facilitates glucose transportation by GLUT2	[18]
Caco-2 cells	AICAR	inhibits PEPT1	[32]
Caco-2 cells	AICAR	promotes ZO-1 assembly and E-cadherin; enhances barrier function; inhibits intestinal permeability	[19]
mice colon	AICAR	promotes goblet cells; inhibits infiltration of inflammatory cells; downregulates macrophages	[33]
Caco-2 cells; mice jejunum; human colonic mucosa	AICAR; metformin	inhibits chloride secretion	[34]
Caco-2 cells	alcohol	inhibits barrier function; disrupts cytoskeleton integrity	[35,36]
HCT116; SW480; LOVO cells; mice colon	berberine	increases mTOR activity and p53 phosphorylation	[37]
Caco-2 cells	butyrate	enhances barrier function; facilitates ZO-1/Occludin redistribution	[38]
T84 cells; mice colon	chitosan oligosaccharide	promotes tight junction assembly; inhibits NF-κB transcriptional activity; prevents the development of aberrant crypt foci	[34,39]
HT-29 cells	curcumin	induces COX-2	[40]
HT-29 cells	combined 5-fluorouracil and genistein	induces COX-2	[41]
Caco-2 cells	Compound C	promotes PEPT1	[42]
HT-29 cells	EGCG	induces COX-2	[20]
mice jejunum	leptin	promotes GLUT2 and GLUT5; decreases SGLT1	[28]
mice jejunum and colon	high-fat diet	induces PPAR; triggers β-catenin activity; Increases intestinal tumorigenesis and villus length	[43,44]
pig jejunum and ileum	lipopolysaccharide	decreases oleic acid, glutamine and glucose in enterocytes	[45]
IL-10^−/−^ mice colon	metformin	inhibits inflammatory cytokines and DSS-induced acute colitis	[21]
COLO205 cells	metformin	inhibits IL-8 expression and NF-κB transcriptional activity	[21]
rat small intestine	metformin	promotes GLUT5 expression	[46]
mice jejunum	metformin	facilitates localization of GLUT2 to apical membrane	[18]
HCT116 xenografts	metformin	inhibits tumour growth lacking P53	[47]
rat caecum	metformin	increases short chain fatty acid-producing bacteria	[48,49]
rat duodenum	metformin	triggers GLP-1 from enteroendocrine L-cells; activates AMPK in hepatocytes in a non-autonomous manner	[50]
Caco-2 cells	MIYAIRI 588	promotes ZO-1	[51]
Pig jejunum	n-3 polyunsaturated fatty acids	promotes glucose uptake	[52,53]
db/db mice colon	pitavastatin	inhibits colonic preneoplastic lesions	[54,55]
mice colon	phenformin	inhibits chloride secretion	[54,56]
Caco-2 cells	propolis polyphenol	promotes tight junctions; enhances the barrier function	[56,57]
HT-29 cells	plumbagin	induces apoptosis via p53	[57,58]
HT-29 cells	quercetin	induces apoptosis via p53	[58,59]
HT-29 cells	selenium	induces COX-2	[59,60]
Caco-2 cells	theaflavins	inhibits PEPT1	[42,60]
HT-29 cells	20(S)-ginsenoside Rg3	induces apoptosis via p53	[42,61]
pig jejunum and ileum	α-ketoglutarate	stimulates oxidation of energy substrates	[45,61]

## 3. AMPK and intestinal absorption

The intestinal epithelium is composed of microvilli, villi and mucosal folds. To generate net influx, nutrients need to pass through the apical membrane of intestinal cells. Nutrients entering intestinal epithelial cells are either used by these cells or pass through the basolateral membrane of intestine cells into the circulatory system. The apical or basolateral transportation can be energy-dependent (active transport with a carrier) or independent (passive transport). The passive transportation depends on a concentration gradient. However, most of the sugars, amino acids, vitamins and minerals are transported by carriers or their respective transporters [69]. Thus, the functional regulation of intestinal transporters is critical for nutrient transportation.

**Figure 1.**
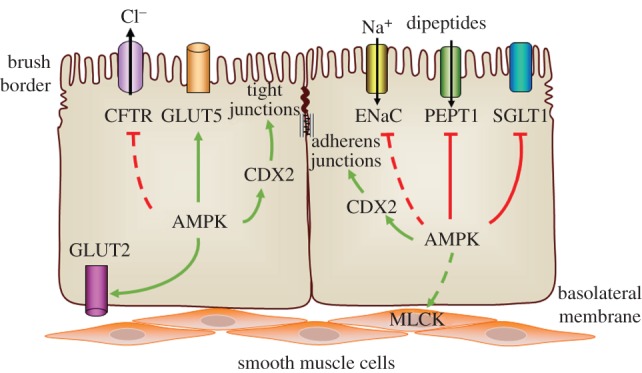
AMPK promotes intestinal absorption and barrier function. AMPK regulates glucose absorption via enhancing the function of glucose transporter (GLUT)2 and GLUT5, while inhibiting sodium–glucose transporter 1 (SGLT1). AMPK mediates ion absorption through possible inhibition of cystic fibrosis transmembrane regulator (CFTR) and epithelial Na^+^ channel (ENaC). Peptide transporter 1 (PEPT1) expression is attenuated by AMPK to reduce apical dipeptide uptake. In addition, AMPK may phosphorylate myosin light chain kinase (MLCK) to enhance vasodilatation and blood flow, further favouring intestinal absorption. Besides absorbing nutrients, the intestine also functions as a frontier barrier protecting the mucosal integrity. AMPK facilitates the establishment of paracellular junctions (tight junctions and adherens junctions) via caudal type homeobox 2 (CDX2), an intestinal transcription factor to upregulate intestinal differentiation. Green arrows indicate positive effects. Red lines indicate negative effects. Solid lines represent proven regulations, while dashed lines represent possible regulations.

Glucose is one of the most important nutrients in our body. Intestinal glucose absorption is mediated by glucose transporters, including glucose transporter 2 (GLUT2), glucose transporter 5 (GLUT5) and sodium–glucose transporter 1 (SGLT1) [70]. The temporal and quantitative regulation of glucose transporters governs glucose flux in and out of the intestine [71]. AMPK regulates glucose uptake not only in the heart [72], skeletal muscle [73], liver [74] and hippocampal neurons [75], but also in the gut. AMPKα2 knockout (KO) decreases protein levels of GLUT2 and GLUT5, while it increases protein levels of SGLT1 in the jejunum [28] (figure 1). Metformin increases GLUT5 expression [46], leading to translocation of GLUT2 to the apical membrane [18], which enhances glucose absorption in the gut [76,77]. Similarly, AICAR inhibits SGLT1 and promotes GLUT2 translocation in mice jejunal mucosa [18]. AMPK is upregulated in rats and pigs by feeding n-3 polyunsaturated fatty acids, which thereafter improve glucose uptake [52,53].

Ion transporters in intestinal epithelium are critical in keeping ion homeostasis [78]. Ion imbalance leads to diarrhoea. AMPK plays an important role in maintaining this homeostasis. Loss of AMPKα1 enhances epithelial sodium (Na^+^) channel (ENaC) expression, which controls the reabsorption of Na^+^ [79] (figure 1). After blocking ubiquitin ligase or endocytosis, phenformin is unable to block ENaC activity, suggesting that ubiquitin ligase is crucial in mediating the effects of AMPK on ENaC ubiquitination [79]. By activating AMPK with AICAR and phenformin in lung epithelial cells, ENaC expression is downregulated, which may be derived from the adaptation to metabolic stress to limit energy dissipation [80]. Apart from ENaC, AMPK also inhibits chloride (Cl^−^) secretion [56], as indicated by the reduction of ion-transport proteins and the cystic fibrosis transmembrane regulator (CFTR) [81] (figure 1). The overstimulation of Cl^−^ secretion by CFTR is the predominant aetiology for enterotoxigenic diarrhoea [82]. AICAR and metformin, which activate AMPK, can offset the increased Cl^−^ efflux by cholera toxin in excised intestinal loops [34], thus preventing diarrhoea. These studies suggest strong clinical applications for AMPK as a potential pharmacological target for treating acute diarrhoeal disease.

Peptide transporters mediate amino acid absorption [83]. AICAR attenuates the expression of peptide transporter 1 (PEPT1) [32], while the AMPK inhibitor Compound C promotes peptide transportation [42] (figure 1). AICAR inhibits apical dipeptide uptake in Caco-2 cells on trans-well filters [32]. The negative correlation between peptide absorption and AMPK activation might be due to the energy-dependent process of peptide uptake, which is dependent on Na/K-ATPase [84]. Thus, peptide transportation is suppressed under nutrient deprivation, probably accompanied by AMPK-mediated signalling pathways.

Another explanation for absorptive dysfunction due to AMPK inhibition is associated with impaired peristaltic activity of visceral musculature. It has been shown that AMPKα mutation in *Drosophila* impairs the movement of food through the digestive tract, which results in smaller fat body cells, delayed metamorphosis and growth inhibition [85]. As mesenteric circulation is directly proportional to nutrient transportation out of the intestine [86], the regulation of capillary blood flow contributes to intestinal absorption. AMPK stimulates vasodilatation and blood flow by attenuating contraction of vascular smooth muscle [87], possibly due to phosphorylation of myosin light chain kinase (MLCK) [88] (figure 1).

## 4. AMPK and intestinal barrier function

Proper intestinal barrier function plays a critical role in our health. Besides absorbing nutrients and secreting fluid, the intestine also functions as a critical barrier maintaining mucosal integrity, which physically inhibits the penetration of harmful substances from the external environment [89]. Impaired barrier function increases intestinal permeability to cause a leaky gut, predisposing individuals to intestinal bowel disease [90], metabolic syndromes [91–94] and autoimmune disorders [95].

**Figure 2.**
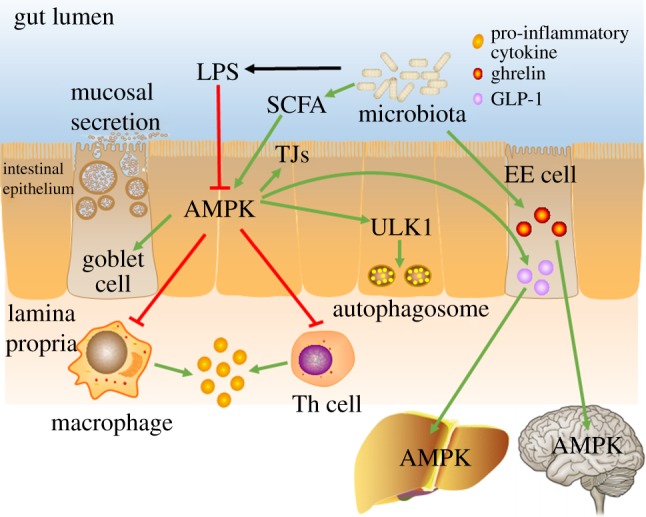
AMPK regulates intestinal inflammation and hormone secretion. AMPK suppresses intestinal inflammation through reducing pro-inflammatory cytokine production in macrophages, inhibiting the differentiation of T helper (Th) cells, promoting mucus secretion and enhancing autophagy, collaboratively. AMPK blocks the secretion of pro-inflammatory cytokines via inhibiting macrophage infiltration and differentiation of Th cells; AMPK triggers autophagy through activation of Unc-51-like autophagy activating kinase 1 (ULK1); AMPK increases goblet cells and associated mucus section, and enhances tight junctions (TJs) to strengthen intestinal barrier function. Gut microbiota and their metabolites such as short-chain fatty acids (SCFAs) regulate AMPK activation, exerting beneficial effects. Additionally, gut microbiota induces enteroendocrine (EE) cells to generate the gut hormone ghrelin, leading to AMPK activation in the hypothalamus to increase food intake. On the other hand, the microbiota upregulates glucagon like peptide 1 (GLP-1) production from EE cells, which augments AMPK phosphorylation in the liver, subsequently reducing hepatic glucose production. Green arrows indicate positive effects; red lines indicate negative effects.

The major determinant of gut epithelial permeability is closure or opening of paracellular junctions between enterocyte intercellular spaces [89]. The gaps between adjacent cells are mechanically sealed by junctional complexes, including desmosomes, adherens junctions (AJs) and tight junctions (TJs) [96]. Tight junctions contribute to the selective paracellular permeability, while AJs are essential for TJ assembly [97]. Thus, intestinal barrier function depends on the performance of intestinal paracellular junctions, such as the establishment and reassembly of TJs, which is regulated by AMPK (figure 2).

In AMPK kinase-dead MDCK cells and Caco-2 cells, the formation of TJs (ZO-1) and establishment of transepithelial electric resistance (TEER) are delayed after calcium switch, while AICAR accelerates TJ reassembly [19,98]. Consistently, intestinal epithelium-specific deletion of AMPK α1 (AMPK VilCre) in mice enhanced intestinal permeability [19] (figure 1). Transmission electron microscopic observation further indicates that the ultrastructure of TJs is less compact in AMPK VilCre mice [19]. The paracellular junction is developed during intestinal differentiation [99], promoted by the intestinal transcription factor caudal type homeobox 2 (CDX2) [100]. AMPK promotes the expression of CDX2 and changes the CDX2 promoter-specific epigenetic modifications [19], providing a possible regulatory mechanism of AMPK on intestinal barrier function (figure 1). Interestingly, AMPK could be activated during TJ assembly via calcium switch, possibly due to the stimulated CaMKK (an AMPK activator) by an influx of calcium [98]. Furthermore, rapamycin rescues the delay of TJ assembly in AMPK kinase-dead cells, demonstrating that AMPK may, at least partially, mediate junction assembly via mammalian target of rapamycin (mTOR) signalling [98]. Additionally, AMPK profoundly promotes the formation of TJs in renal [101], mammary [102] and hepatic [103] epithelial cells.

Many environmental factors impact intestinal barrier function associated with alteration of AMPK. Chitosan oligosaccharide and polyphenol extracts from Propolis upregulates TJ assembly and enhances the barrier function associated with AMPK activation [39,57]. The consumption of alcohol, a potent metabolic stressor, diminishes cellular ATP production and increases intestinal permeability [35,36]. AMPK activators such as metformin and AICAR exert ameliorative effects on disrupted barrier function caused by bacterial [104] and viral pathogens [105], and pro-inflammatory cytokines [106]. Epithelial barriers are dysfunctional in AMPK VilCre mice [19], while metformin supplementation suppresses intestinal permeability, probably due to augmentation of epithelial differentiation [67].

Microbial metabolites, short-chain fatty acids (SCFAs), activate AMPK in colonocytes [38], associated with the enhanced TEER and redistribution of TJs [38]. Furthermore, butyrate protects against ethanol-induced intestinal barrier dysfunction, accompanied with AMPK activation [36]. *Clostridium butyricum* MIYAIRI 588, the butyrate-producing probiotic, enhances the activity of AMPK and strengthens TJs, resulting in mitigated gut permeability in non-alcoholic fatty liver disease [38,51]. Inhibition of AMPK either genetically or chemically abolishes the aforementioned therapeutic or prophylactic potential of SCFAs, demonstrating the modulatory role of AMPK in SCFAs’ enhanced barrier function [36].

## 5. AMPK and intestinal inflammation

The pathology of intestinal inflammation is mainly associated with immunological disorders [107]. Immune cells produce pro-inflammatory cytokines to defend against bacterial infections [108], triggering the activation of T cells and the recruitment of neutrophils [109]. Deficient regulatory T cells, excessive effector T cells and the overproduction of pro-inflammatory cytokines are all prone to inducing intestinal inflammation that exacerbates colitis [110,111]. Thus, the blocking of pro-inflammatory cytokines provides a therapeutic potential for inflammatory bowel disease (IBD), an autoimmune disease of the intestine [112].

Intestinal macrophages in the lamina propria are the primary source of pro-inflammatory cytokines [108]. The expression of inducible nitric oxide synthases (iNOSs) and tumour necrosis factor (TNF)α and phosphorylation of nuclear factor-kappa B (NF-κB) are inhibited in intestinal mucosal macrophages treated with AICAR [33]. *In vitro*, metformin suppresses TNFα-induced IL-8 expression and NF-κB inflammatory signalling [21], which facilitates T-cell differentiation [113]. AICAR suppresses the differentiation of Th1 and Th17 cells, possibly due to the downregulation of their transcriptional factors, T-bet and RORγt [33]. Lipopolysaccharides (LPSs), the major outer membrane component of Gram-negative bacteria, contribute to the inflammatory process of IBD [114]. LPS administration decreases phosphorylation of AMPK in pig jejunum and ileum (figure 2), which is ameliorated by dietary supplementation with α-ketoglutarate [45]. In addition, intraperitoneal injection with AICAR downregulates colonic macrophage activation in LPS-induced or 2,4,6-trinitrobenzenesulfonic acid (TNBS)-induced colitis [33]. Thus, AMPK may provide an intervening target to ameliorate LPS-induced gut damage.

The mucus layer produced by goblet cells provides an additional protective barrier to the gut epithelium. The defective formation of the mucus layer increases mouse susceptibility to colitis [115], and colitis reduces the size and number of goblet cells in human gut [116]. AICAR supplementation augments goblet cell differentiation and attenuates the infiltration of inflammatory cells in TNBS-induced acute colitis [33] (figure 2).

The immune defences and repair systems are activated to maintain tissue integrity upon pathogen infection. Autophagy enhances cell survival under stress conditions, and keeps the balance between immunity and inflammation. This may play a protective role against inflammatory diseases such as IBD [117]. Unc-51-like autophagy activating kinase 1 (ULK1) is the earliest trigger for autophagocytosis, which is phosphorylated and binds with mTORC1 when nutrients are sufficient [118]. When nutrients are deprived, ULK1 dissociates from mTORC1, subsequently leading to initiation of autophagy [118]. AMPK involves ULK1-engaged autophagy by directly phosphorylating ULK1 at Ser 317 and Ser 777 to initiate autophagy [119] (figure 2).

Inflammation is closely related to metabolism. To synthesize ATP, cells can undergo either glycolysis or aerobic oxidation. Inflammatory cells, such as macrophages and T helper (Th) cells, typically undergo glycolytic metabolism [120]; on the other hand, anti-inflammatory cells typically depend on oxidative metabolism through mitochondria [120]. AMPK activation creates a pseudo-starving state that promotes oxidative metabolism and inhibits inflammation [120]. Similarly, creatine can regulate metabolism by recycling ATP in cells. Mutation in the creatine biosynthesis enzyme increases mice colitis, while creatine supplementation ameliorates colitis, possibly related to ATP supply and AMPK activation [121]. Collectively, those studies suggest that AMPK regulates intestinal inflammation partially through altering cell metabolism. AMPK activation shifts pro-inflammatory to anti-inflammatory cytokine production in macrophages, facilitates the differentiation of Th cells, and promotes epithelial barrier function and epithelial autophagy.

Up to now, most studies on the effects of AMPK on inflammation have been short-term studies. Limited information also points to the long-term effects of AMPK in suppressing inflammation. In an epidemiologic study, metformin suppresses chronic inflammation as indicated by the ratio of neutrophils to lymphocytes in the blood sample of diabetic patients [122]. Considering the commonness of chronic intake of AMPK activators, such as phenformin and metformin in diabetic patients, the long-term effect of AMPK activation on inflammation needs to be further investigated.

## 6. AMPK and colorectal cancer

Intestinal epithelium is the most dynamic tissue in the body, as it is constantly being renewed. Perturbations of the balance among proliferation, differentiation and apoptosis could result in the predisposition to and initiation of colorectal cancer (CRC), the third most lethal cancer in the United States [123,124]. Unlike normal epithelial cells, cancer cells depend on glycolysis to provide energy, the so-called Warburg effect [125]. As a metabolic mediator, AMPK is an anti-apoptotic component when cells are injured from glucose deprivation [126], hyperglycaemia [127], ceramide production [128] and ischaemia [129]. In contrast, AMPK induces apoptosis in pancreatic cells [130], gastric cancer cells [131] and neuroblastoma cells [132]. Thus, the effects of AMPK on apoptosis are dependent on cell type and external stimuli.

AMPK activation in the intestine is inhibited in metabolic diseases. In rats with insulin resistance, the protein content of phospho-AMPK is markedly decreased in the jejunum [24]. A high-fat diet (HFD) inactivates AMPK, and triggers β-catenin activity due to the induction of the peroxisome proliferator-activated receptor, which thereafter increases intestinal tumorigenesis and villus length [43,44]. Pitavastatin has chemopreventive potential on obesity-related colorectal carcinogenesis, associated with AMPK activation [54]. The adiponectin secreted by adipose tissue links metabolic diseases to tumorigenesis, probably mediated through AMPK. The incidence of colonic polyps increases in colorectal cancer with adiponectin deficiency, concomitantly with the inactivation of AMPK [133]. Consistently, growth inhibition by adiponectin in HT-29 cells is attenuated due to AMPK deficiency, and promoted when metformin is presented [30].

**Figure 3.**
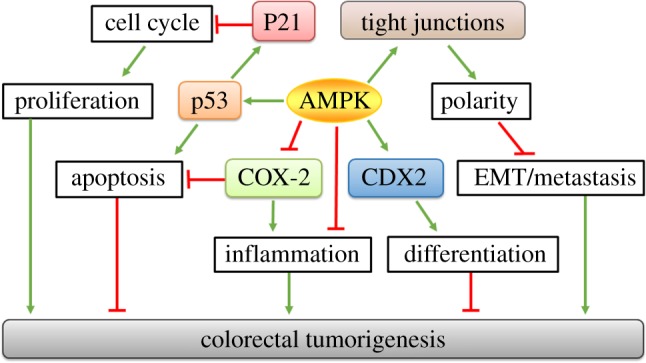
Suppressive mechanisms of AMPK in colorectal tumorigenesis. AMPK may suppress colorectal tumorigenesis through inhibiting proliferation, inflammation and metastasis as well as promoting apoptosis and differentiation collaboratively. AMPK induces apoptosis via p53 activation, which further upregulates p21 to impede cell cycling progression and cell proliferation; AMPK inhibits cancer progression by promoting caudal type homeobox 2 (CDX2) to induce differentiation, and by abating cyclooxygenase-2 (COX-2) to regulate apoptosis and inflammation; AMPK enhances the establishment of tight junctions to form epithelial polarity, which subsequently results in the amelioration of epithelial–mesenchymal transition (EMT) and colorectal metastasis. Green arrows indicate positive effects; red lines indicate negative effects.

Mechanistically, AMPK could inhibit colorectal carcinogenesis through upregulating p53, a tumour suppressor (figure 3). Quercetin [59], the major dietary flavonoid, 20(S)-ginsenoside Rg3 from Panax ginseng [61] and plumbagin from plants [58] cause apoptosis via an AMPK–p53 cascade. Oral administration of quercetin reduces tumour volume and activates AMPK in mice with HT-29 tumour xenografts [59]. Activated AMPK phosphorylates p53 at Ser 15, which is partially responsible for the inhibition of tumour growth [134]. Furthermore, berberine activates AMPK, decreases mTOR activity and phosphorylates p53 in HCT116 cells [37]. Similar effects of quercetin were also detected in lung [135], breast [136], leukaemia [137] and prostate cancer cells [138]. As a tumour suppressor, p53 is often mutated in cancer. Of note, metformin and AICAR selectively inhibit tumour growth in HCT116 xenografts lacking p53, but not in those with intact p53 [139]. This may suggest a potential anti-tumour effect of metformin in patients with p53-deficient tumours.

AMPK could also inhibit colorectal cancer through inhibiting cyclooxygenase-2 (COX-2), an inflammatory enzyme (figure 3). Epigallocatechin gallate (EGCG), a polyphenol derived from green tea, stimulates AMPK in a dose-dependent manner [20]. The activated AMPK thereafter inhibits the production of COX-2 and prostaglandin E2 to induce apoptosis, while Compound C abolishes it [20]. Similarly, curcumin [40], selenium [60], and combined 5-fluorouracil and genistein [41] all demonstrate anti-tumorigenic effects via the AMPK–COX2 cascade.

In addition, AMPK exerts anti-tumour effects through arresting the cell cycle and inducing apoptosis (figure 3). Adiponectin stimulates AMPK, arrests cell cycle progression at the G_1_ phase, reduces cyclin E, and stimulates p21, p27, glucose utilization and fatty acid oxidation [31]. AMPK arrests the cell cycle to inhibit proliferation in many established cancer cells including prostate cancer PC-3 [140], hepatoma HepG2 [141], brain C6 glioma, astrocytoma U87MG, T-lymphoblast CEM and breast cancer MCF-7 [142] cells.

CDX2 overexpression inhibits the growth and proliferation of colorectal cancer cells [143,144]. CDX2 expression is dramatically decreased during the late stages of malignant colorectal cancer [145]. CDX2 is absent in 183 out of 621 colorectal cancers from patient specimens [146]. Its loss is positively correlated with tumour grade and stage [146]. AICAR treatment upregulates CDX2 expression in Caco-2 cells, while CDX2 expression is dramatically downregulated in AMPKα2 KO Caco-2 cells and AMPK VilCre KO mice [19] (figure 3). CDX2 deletion abolished the promoted effects of AMPK on intestinal differentiation markers [19]. CDX2 mutation upregulates colonic polyp number and increases the proliferation of colonic cells [147], whereas the re-expression of CDX2 inhibits cyclin D1 expression and cell proliferation in human intestinal epithelial crypt cells lacking *Cdx2* [148]. As CDX2 is a transcription factor that facilitates intestinal differentiation, enhancing differentiation could be a promising strategy for anti-cancer therapy.

The loss of epithelial polarity leads to the damage of intestinal organization, which is probably the major step for neoplastic transformation [149], subsequently resulting in epithelial–mesenchymal transition and metastasis [150]. It has been shown that AMPKα mutation in *Drosophila* embryos leads to abnormal distribution of epithelial polarity markers [151]. The consequent loss of polarity along with over-proliferative aberration elicits tumorous growths [152,153]. Therefore, the strengthened tight junction by AMPK provides a possible method for inhibiting adenocarcinoma and tumorigenesis (figure 3).

Chronic inflammation dramatically increases the risk of tumorigenesis. The reactive nitrogen intermediates and reactive oxygen species associated with inflammation usually trigger genomic instability and induce genetic mutations [154]. The DNA damage in turn initiates colorectal carcinogenesis. Intestinal inflammation is strongly associated with colon cancer, which has been comprehensively discussed by Terzić and co-authors [155]. AMPK suppresses many aspects of intestinal inflammation, which is discussed in §5 ‘AMPK and intestinal inflammation’. AMPK VilCre KO mice demonstrate exacerbated dextran sodium sulfate (DSS)-induced colitis [19], while metformin administration reduces colitis in interleukin-10-deficient mice [67] as well as DSS-induced colitis in mice [21]. AMPK might inhibit intestinal tumorigenesis through mitigating intestinal inflammation (figure 3).

## 7. Gut microbiota regulates AMPK activity

Gut microbiota show a close relationship with intestinal health [156]. Metagenomic analysis shows that the populations of Firmicutes and Bacteroidetes are profoundly reduced in the gut microbiota from IBD patients [157]. Bifodobacteria, *Lactobacillus* and *Bacteroides* ameliorate IBD, while *Helicobacter hepaticus* exacerbates IBD [156], probably due to their difference in SCFA production. SCFAs activate AMPK in colonocytes; both venous infusion and oral administration of SCFAs to mice activate AMPK [158], which may explain the regulatory effect of gut microbiota on AMPK activity (figure 2). Oral administration of metformin or berberine increases the population of *Allobaculum*, *Bacteroides*, *Blautia*, *Butyriciococcus* and *Phascolarctobacterium* in gut microbiota, which promotes SCFA production [48].

Besides activation by low energy level, AMPK can also be regulated by intestinal hormones in a cell non-autonomous manner [159,160] (figure 2). Prebiotic treatment enhances the generation of gut hormones, glucagon-like peptide (GLP)-1 and GLP-2, due to an increase in enteroendocrine L-cells in the colon of obese mice [161] (figure 2). Likewise, metformin triggers L-cells in rat duodenum to secrete GLP-1 [50]. GLP-1 enhances AMP and subsequently activates AMPK in hepatocytes to reduce hepatic glucose production in a non-autonomous manner [162]. However, AMPK mutation in hepatocytes abolished the beneficial effects of the gut-derived peptide GLP-1 [162].

Constant activation of AMPK in the hypothalamus is able to increase food intake and body weight, while AMPK inactivation reduces appetite in rodents [163,164]. Intraperitoneal injection of ghrelin, the appetite-stimulating gastrointestinal hormone, upregulates rat food intake, associated with AMPK activation in presynaptic neurons [164,165] (figure 2). Consistently, AICAR injection into rat hypothalamus or cerebral ventricle enhances food intake [164], suggesting the integrative effect of AMPK in whole body metabolic regulation.

## 8. Conclusion

**Figure 4.**
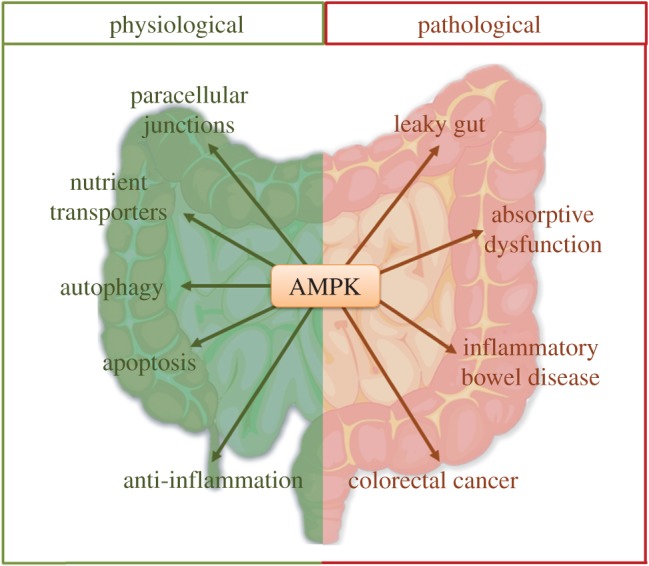
Implications and therapeutic perspectives of AMPK in the intestine. Under physiological conditions, AMPK activation strengthens paracellular junctions, enhances the function of nutrient transporters, promotes autophagy and apoptosis, and exerts anti-inflammatory effects. On the other hand, under pathological conditions, AMPK inactivation is implied in a number of intestinal diseases, such as leaky gut, absorptive dysfunction, inflammatory bowel disease and colorectal cancer.

AMPK exerts protective effects on intestinal epithelial function through multiple mechanisms including improving intestinal absorption, enhancing barrier function, suppressing inflammation and preventing colorectal cancer. AMPK activation, either by pharmacological means or by nutraceuticals, might be a promising therapeutic strategy for treatment of intestinal disorders (figure 4).

## Data accessibility

This article has no additional data.
